# 1‑(Phenylselanyl)-2‑(*p*‑tolyl)indolizine Mitigates Lipopolysaccharide (LPS)-Induced Depressive-Like Behavior by Modulating Oxidative Stress and Inflammatory Markers

**DOI:** 10.1021/acsomega.5c11651

**Published:** 2026-01-20

**Authors:** Marcia J. da Rocha, Marcelo H. Presa, Gustavo D. Nunes, Victor S. Barboza, Janice L. Giongo, Rodrigo A. Vaucher, Nathalia S. Pedra, Roselia M. Spanevello, Francieli M. Stefanello, Caroline S. Gomes, Eder J. Lenardão, Filipe Penteado, Cristiani F. Bortolatto, César A. Brüning

**Affiliations:** † Laboratory of Biochemistry and Molecular Neuropharmacology (LABIONEM), Graduate Program in Biochemistry and Bioprospecting (PPGBBio), Chemical, Pharmaceutical and Food Sciences Center (CCQFA), 37902 Federal University of Pelotas (UFPel), P.O. Box 354, Pelotas, RS 96010-900, Brazil; ‡ Laboratory of Research in Biochemistry and Molecular Biology of Microorganisms, Graduate Program in Biochemistry and Bioprospecting, Federal University of Pelotas (UFPel), P.O. Box 354, Pelotas, RS 96010-900, Brazil; § Biomarker Laboratory, Graduate Program in Biochemistry and Bioprospecting, Federal University of Pelotas (UFPel), P.O. Box 354, Pelotas, RS 96010-900, Brazil; ∥ Clean Organic Synthesis Laboratory (LASOL), Graduate Program in Chemistry (PPGQ), Chemical, Pharmaceutical and Food Sciences Center (CCQFA), Federal University of Pelotas (UFPel), P.O. Box 354, Pelotas, RS 96010-900, Brazil; ⊥ Department of Chemistry, Federal University of Santa Maria − UFSM, Av Roraima, Building 18, Santa Maria, RS 97105-900, Brazil

## Abstract

1-(phenylselanyl)-2-(*p*-tolyl)­indolizine (MeSeI) is a selenoindolizine that showed antidepressant-like properties in mice via monoaminergic and glutamatergic systems. This study aimed to investigate the MeSeI effect on lipopolysaccharide (LPS)-induced depressive-like behavior in mice as well as its effect on oxidative stress parameters in primary astrocyte cultures challenged with LPS. Primary astrocyte cultures were exposed to LPS (1 μg/mL) for 3 h and treated with MeSeI (5, 10, 15, 25 μM) for 48 h. MeSeI reversed the LPS-induced increase in reactive species (RS) and nitrite levels, restored the activity of antioxidant enzymes, and increased the sulfhydryl content in astrocytes. Male *Swiss* mice received MeSeI (10 mg/kg, intragastrically) 30 min prior to LPS administration (0.83 mg/kg, intraperitoneally). After 24 h, the animals were subjected to behavioral tests and then euthanized to remove the prefrontal cortex (PFC) and plasma. MeSeI prevented LPS-induced depression-like behavior, the increase in NF-κB and IL-6 expression, and IL-6 protein levels, as well as RS levels and lipid peroxidation in the PFC, and reduced plasma corticosterone levels induced by LPS. These findings suggest that MeSeI exerts neuroprotective effects by modulating the neuroinflammatory pathway and normalizing oxidative stress parameters, indicating its potential as a therapeutic candidate for depression treatment.

## Graphical abstract

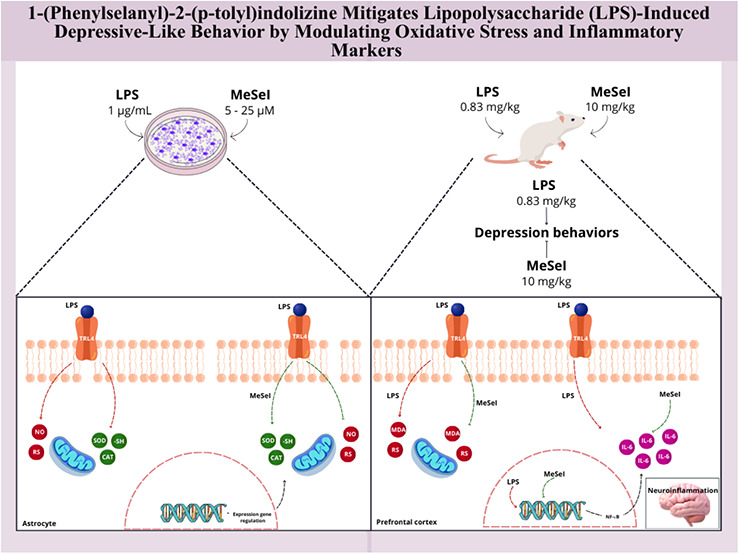

## Introduction

Depression is a serious mental illness, mainly characterized by large mood swings and cognitive function disorders.^1^ According to the World Health Organization (WHO),^1^ depression affected 280 million people worldwide in 2019, and the therapeutic effect of antidepressants is currently less than 70%.^2^ The COVID-19 pandemic resulted in approximately 53.2 million additional cases of depression in 2020, representing a 27.6% surge in prevalence.^3^ Therefore, it is imperative to develop new and highly effective antidepressants.

Increasing evidence indicates that neuroinflammation plays a vital role in the development of depression in humans and preclinical animal studies.^4–6^ Neuroinflammation is associated with the activation of microglia and astrocytes in the central nervous system (CNS), promoting the excessive release of pro-inflammatory cytokines, leading to the inhibition of neurogenesis and impaired synaptic plasticity.^7,8^ Furthermore, neuroinflammation is associated with increased oxidative damage, especially in the prefrontal cortex (PFC).^9^ Taken together, these factors could potentially lead to the development of depressive symptoms.

The lipopolysaccharide (LPS) response model is a recognized animal model for studying depression and inflammation.^10^ Previous studies have reported that depression-like behaviors can be observed 24 h after LPS treatment, and the immune system is activated by its interaction with toll-like receptor (TLR)-4 in glial cells.^11^ LPS can elicit astrocytic reactivity, which induces the secretion of elevated levels of pro-inflammatory cytokines, exacerbates oxidative stress, and disrupts neuronal excitability and synaptic plasticity.^8^ Therefore, suppressing neuroinflammation and astrogliosis may offer a promising alternative strategy for treating depression.

Recently, our group demonstrated that 1-(phenylselanyl)-2-(*p*-tolyl)­indolizine (MeSeI) presents antidepressant-like action by monoaminergic and glutamatergic modulation.^12–14^ MeSeI is a small-molecular-weight hybrid of an indolizine nucleus and selenium that demonstrates *in vitro* antioxidant activity.^15^ Furthermore, many studies have demonstrated the anti-inflammatory and antidepressant-like effects of molecules containing an indolizine nucleus^16,17^ and organoselenium compounds.^18^ Thus, this study aimed to investigate the neuroprotective and anti-inflammatory effects of MeSeI using LPS-stimulated primary astrocyte cultures and LPS-induced depression-like behavior in mice to determine the mechanism of action of MeSeI in neuroinflammation.

## Results and Discussion

In the present study, we demonstrated that MeSeI could reverse LPS-induced oxidative stress and enhance cellular antioxidant defense in an astrocyte culture. In addition, we demonstrated the effect of MeSeI on neuroinflammation induced by LPS and its potential mechanisms. MeSeI ameliorated depression-like behaviors in mice by decreasing the immobility time in the forced swimming test (FST) and tail suspension test (TST), and by increasing the grooming activity time in the splash test (ST). Furthermore, MeSeI treatment significantly decreased reactive species (RS) and lipid peroxidation induced by LPS in PFC and prevented an increase in plasma corticosterone levels. Additionally, MeSeI treatment reduced the nuclear factor-kappa B (NF-κB) and IL (interleukin)-6 expression as well as IL-6 protein levels in LPS-induced PFC. Taken together, these findings suggest that MeSeI can significantly alleviate depression by attenuating neuroinflammation and decreasing oxidative stress parameters.

### MeSeI Ameliorated Oxidative Stress in LPS-Induced Neuroinflammation in Primary Astrocyte Culture

Astrocytes have been recognized for their significant roles in regulating neuroinflammation and supporting neuroprotection. These cells make up about 30% of the central nervous system (CNS) and are vital for various physiological functions, including metabolic balance and the release of gliotransmitters.^19^ Thus, the astrocytes are considered as a possible target for new therapeutic approaches for a variety of CNS disorders, including depression.^20,21^

In this context, new therapeutic approaches for depression must ensure safety and exhibit a low toxicity. Therefore, to ensure that MeSeI treatment does not affect astrocytes, we assessed the viability and proliferation by 3-(4,5-dimethylthiazol-2-yl)-2,5-diphenyl tetrazolium bromide (MTT) and sulforhodamine B (SRB) assays, respectively. As shown in Figure 1a, MeSeI at 5 to 25 μM concentrations did not change astrocyte cell viability compared to vehicle cells even after 48 h of treatment (*F*_4, 10_ = 1.063, *p* < 0.4237). However, the results presented in Figure 1b show that MeSeI significantly increased astrocyte cell proliferation compared to vehicle cells following 48 h of treatment (*F*_4, 10_ = 23.99, *p* < 0.0001). A previous study carried out by our research group demonstrated that MeSeI presents low toxicity potential in female *Swiss* mice at a dose of 300 mg/kg.^12^ In this study, the authors demonstrated that the administration of MeSeI at a dose of 300 mg/kg did not result in mortality in any of the animals during the experimental protocol and did not affect food or water intake. Furthermore, biochemical analyses revealed that treatment with MeSeI did not change plasma aspartate and alanine aminotransferases (AST and ALT, respectively) or urea levels when compared with the control group. Taken together, these findings suggest that MeSeI exhibits a low toxicity potential profile as a promising antidepressant compound.

**Figure 1:**
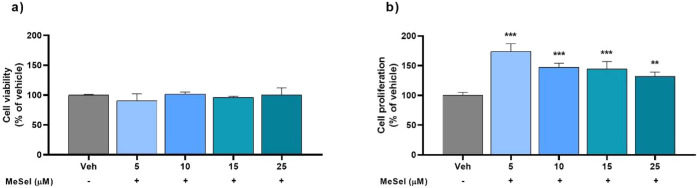
Effect of MeSeI on primary astrocytic culture viability (*F*_4, 10_ = 1.063, *p* < 0.4237) (a) and proliferation (*F*_4, 10_ = 23.99, *p* < 0.0001) (b) following 48 h of treatment (*n* = 3 independent experiments, each one performed in quadruplicate). The values were represented as the mean ± SD and were analyzed using a one-way ANOVA followed by Newman–Keuls post hoc test. (**) *p* < 0.01 and (***) *p* < 0.001 when compared with vehicle group. Abbreviations: Veh—vehicle; MeSeI—1-(phenylselanyl)-2-(*p*-tolyl)­indolizine.

Astrocytes respond to this inflammation through a process called astrocyte reactivity, which is characterized by cellular hypertrophy. This reaction leads to increased extracellular glutamate levels and further intensifies the neuroinflammatory state by generating pro-inflammatory cytokines. Additionally, it can cause neuronal death through the production of toxic levels of RS and nitric oxide (NO).^8^ As the astrocyte reactivity and glutamatergic system are linked, we previously showed that MeSeI exerts antidepressant-like action by modulating the *N*-methyl-d-aspartate (NMDA) receptor,^14^ raising the hypothesis that its effect also occurs through astrocyte regulation.

As demonstrated in this study, LPS (1 μg/mL) induced a significant increase in the levels of RS (Figure 2a; *F*_5, 12_ = 31.20, *p* < 0.0001) and nitrites (Figure 2b; *F*_5, 12_ = 5.954, *p* = 0.0054) and reduced the SH content in the astrocyte cells (Figure 2c; *F*_5, 12_ = 12.21, *p* = 0.0002) after 48 h. The MeSeI treatment at concentrations of 5, 10, 15, and 25 μM reversed this increase in RS and nitrite levels, and the MeSeI compound at a concentration of 25 μM was able to increase the level of SH content, demonstrating its protective effect against oxidative stress in astrocytes (Figure 2a–c). In the thiobarbituric acid reactive species (TBARS) assay, LPS induced significant lipid peroxidation, and the compound reduced oxidative damage after 48 h of incubation (Figure 2d; *F*_5, 12_ = 23.25, *p* < 0.0001), further supporting its protective effect on these cells.

**Figure 2:**
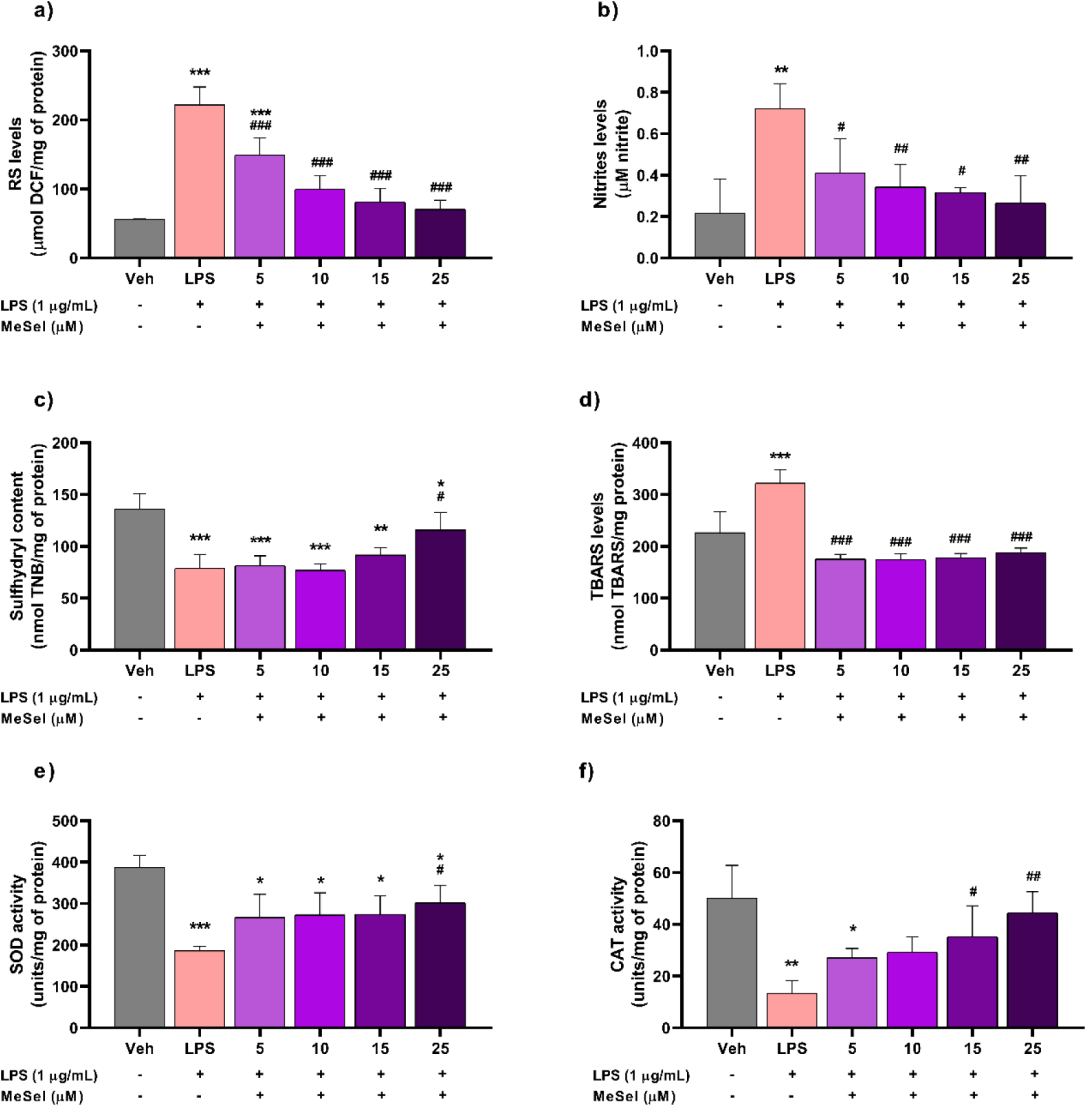
Effect of MeSeI (5, 10, 15, and 25 μM) on RS (*F*_5, 12_ = 31.20, *p* < 0.0001) (a), nitrite (*F*_5, 12_ = 5.954, *p* = 0.0054) (b), sulfhydryl (*F*_5, 12_ = 12.21, *p* = 0.0002) (c), TBARS (*F*_5, 12_ = 23.25, *p* < 0.0001) (d), SOD (*F*_5, 12_ = 7.074, *p* = 0.0027) (e), and CAT (*F*_5, 12_ = 6.898, *p* = 0.0030) (f) levels in astrocyte primary cultures exposed to LPS (1 μg/mL) after 48 h using the reversal protocol (*n* = 3 independent experiments, each one performed in duplicate). The values were represented as the mean ± SD and were analyzed using a one-way ANOVA followed by Newman–Keuls post hoc test. (*) *p* < 0.05, (**) *p* < 0.01, and (***) *p* < 0.001 when compared with the vehicle group. (^#^) *p* < 0.05, (^##^) *p* < 0.01, and (^###^) *p* < 0.001 when compared with the LPS group. Abbreviations: Veh—vehicle; MeSeI—1-(phenylselanyl)-2-(*p*-tolyl)­indolizine; LPS—lipopolysaccharide; RS—reactive species; TBARS—thiobarbituric acid reactive species; SH—sulfhydryl; SOD—superoxide dismutase; CAT—catalase.

Antioxidant enzymatic activities were assessed in astrocytes treated with LPS and MeSeI to evaluate the compound’s potential protective effects against oxidative stress. The results demonstrated a significant reduction in superoxide dismutase (SOD) and catalase (CAT) activities in astrocytes exposed to LPS compared to the vehicle group after 48 h (Figure 2e; *F*_5, 12_ = 7.074, *p* = 0.0027; Figure 2f; *F*_5, 12_ = 6.898, *p* = 0.0030). However, treatment with MeSeI conferred protection against this reduction at concentrations commencing from 15 μM.

MeSeI prevented the LPS-induced reduction in antioxidant enzyme activities, specifically SOD and CAT, reinforcing its antioxidative potential in astrocytes. These antioxidant enzymes are regulated by nuclear factor erythroid 2-related factor 2 (Nrf-2), a neuroprotective transcription factor that modulates several detoxification genes encoding antioxidant proteins, which confer protection against oxidative stress induced by neuroinflammation.^22^ Another enzyme indirectly involved in oxidative stress is monoamine oxidase (MAO). Inhibitors of this enzyme can reduce oxidative stress imbalance and exhibit antidepressant properties.^23^ In this context, MeSeI has been demonstrated to inhibit both MAO-A and MAO-B enzymes *in vitro* and *in vivo*.^13^ Consequently, the assessment of compounds exhibiting anti-inflammatory and antioxidant properties is essential for the treatment and prevention of neuroinflammation associated with depression.

### MeSeI Treatment Exhibits a Neuroprotective Effect in the Preclinical Model of LPS-Induced Depression in Mice

In the LPS animal model of depression, the efficacy of antidepressant-like treatments can be assessed through behavioral paradigms, such as FST, TST, and ST. Treatment with LPS significantly elevated the immobility time in FST (Figure 3a) and TST (Figure 3b) compared with the vehicle group, demonstrating that there was depression-like behavior in the animals. In contrast, these results were prevented in the group treated with MeSeI and LPS, since MeSeI decreased the immobility time in FST (Figure 3a; *F*_4, 40_ = 13.13, *p* < 0.0001) and TST (Figure 3b; *F*_4, 37_ = 12.29, *p* < 0.0001) in mice treated with LPS, according to one-way ANOVA results. These results indicated that MeSeI had an antidepressant-like effect in mice. FLX showed similar results to the MeSeI treatment in the FST and TST in mice.

**Figure 3:**
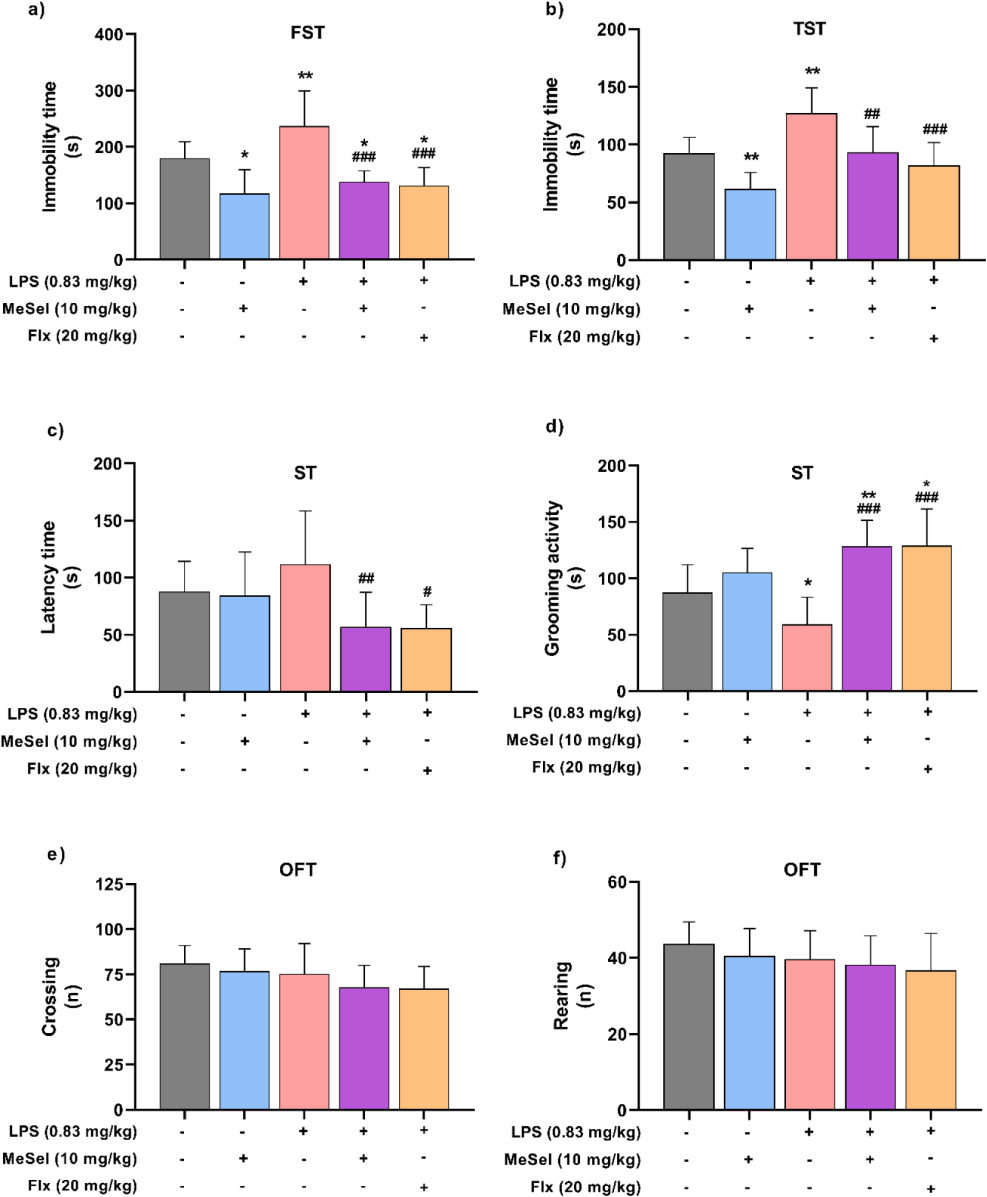
MeSeI mitigated the LPS-induced depression behaviors in mice. Immobility time (*F*_4, 40_ = 13.13, *p* < 0.0001) (a) in FST; immobility time (*F*_4, 37_ = 12.29, *p* < 0.0001) (b) in TST; latency for the first grooming (*F*_4, 37_ = 4.164, *p* = 0.0070) (c) and grooming activity time (*F*_4, 37_ = 10.99, *p* < 0.0001) (d) in ST; number of crossings (*F*_4, 40_ = 1.948, *p* = 0.1213) (e) and number of rearings (*F*_4, 40_ = 1.008, *p* = 0.4149) (f) in OFT (1st set: *n* = 8–10 animals for OFT and FST; 2nd set: *n* = 7–9 animals for TST and ST). The values were represented as the mean ± SD and were analyzed using a one-way ANOVA followed by Newman–Keuls post hoc test. (*) *p* < 0.05 and (**) *p* < 0.01 when compared with vehicle group. (^#^) *p* < 0.05, (^##^) *p* < 0.01, and (^###^) *p* < 0.001 when compared with LPS group. Abbreviations: Veh—vehicle; MeSeI—1-(phenylselanyl)-2-(*p*-tolyl)­indolizine; LPS—lipopolysaccharide; FLX—fluoxetine; OFT—open field test; FST—forced swimming test; TST—tail suspension test; ST—splash test.

In addition, ST results showed that compared to the vehicle group, treatment with LPS tended to increase the latency time to grooming (Figure 3c) and significantly decreased the grooming activity time (Figure 3d). A one-way ANOVA test revealed that the latency time in the interaction group (MeSeI + LPS) was significantly lower than in the LPS one (Figure 3c; *F*_4, 37_ = 4.164, *p* = 0.0070). Furthermore, the grooming activity time in the MeSeI + LPS group was significantly higher than in the LPS one (Figure 3d; *F*_4, 37_ = 10.99, *p* < 0.0001). This data demonstrated that MeSeI can prevent the LPS-induced depression-like behavior in ST in mice. Fluoxetine (FLX) showed similar results to the MeSeI treatment in ST in animals.

The treatment with LPS or MeSeI did not change the crossing (Figure 3e; *F*_4, 40_ = 1.948, *p* = 0.1213) or rearing (Figure 3f; *F*_4, 40_ = 1.008, *p* = 0.4149) numbers in the open field test (OFT), which is consistent with literature showing that LPS treatment does not affect autonomous behavior or motor ability in mice.^24^ In this study, the purpose of evaluating the locomotor activity of mice by OFT was to exclude the interference of any locomotor change caused by the treatments in the immobility time in FST and TST, and self-grooming in ST, confirming that MeSeI has a potent antidepressant effect in LPS-induced depression.

Consistent with previous reports, our results showed that LPS administration evoked depression-like behavior in mice, significantly increasing the time of immobility in FST and TST, and decreasing grooming activity time in ST. In this study, it was observed that MeSeI treatment significantly decreased LPS-induced depressive behaviors in FST, TST, and ST, implying that the compound alleviates depression-like behavior in mice. FLX, a typical selective serotonin reuptake inhibitor (SSRI), was used as a positive control in this experiment and shown to effectively prevent depression-like behaviors.

In the literature, the LPS-induced depression model has been widely utilized to investigate potential antidepressant therapies by neuroinflammation modulation, such as oxidative stress parameters and reducing tumor necrosis factor (TNF)-α, IL-6, and corticosterone levels.^24,25^ Collectively, these studies corroborate our findings, demonstrating the efficacy of the LPS-induced neuroinflammation model as well as the investigation of novel potential antidepressant compounds.

To evaluate the MeSeI antioxidant properties, the RS and TBARS levels were measured in the PFC of LPS-insulted mice. As depicted in Figure 4a, a significant difference was observed in RS levels in the PFC (Figure 4a; *F*_3, 27_ = 5.543, *p* = 0.0043) among groups. As indicated by increased DCF fluorescent intensity, RS significantly increased in the PFC area in the LPS group when compared with the vehicle group. In contrast, MeSeI treatment declined RS levels in the PFC compared with the LPS group. TBARS levels increased significantly in the LPS group compared to the vehicle group in the PFC area, while the MeSeI treatment in the interaction group (MeSeI + LPS) significantly prevented the increase in TBARS levels compared to the LPS group in the PFC (Figure 4b; *F*_3, 27_ = 11.68, *p* < 0.0001).

**Figure 4:**
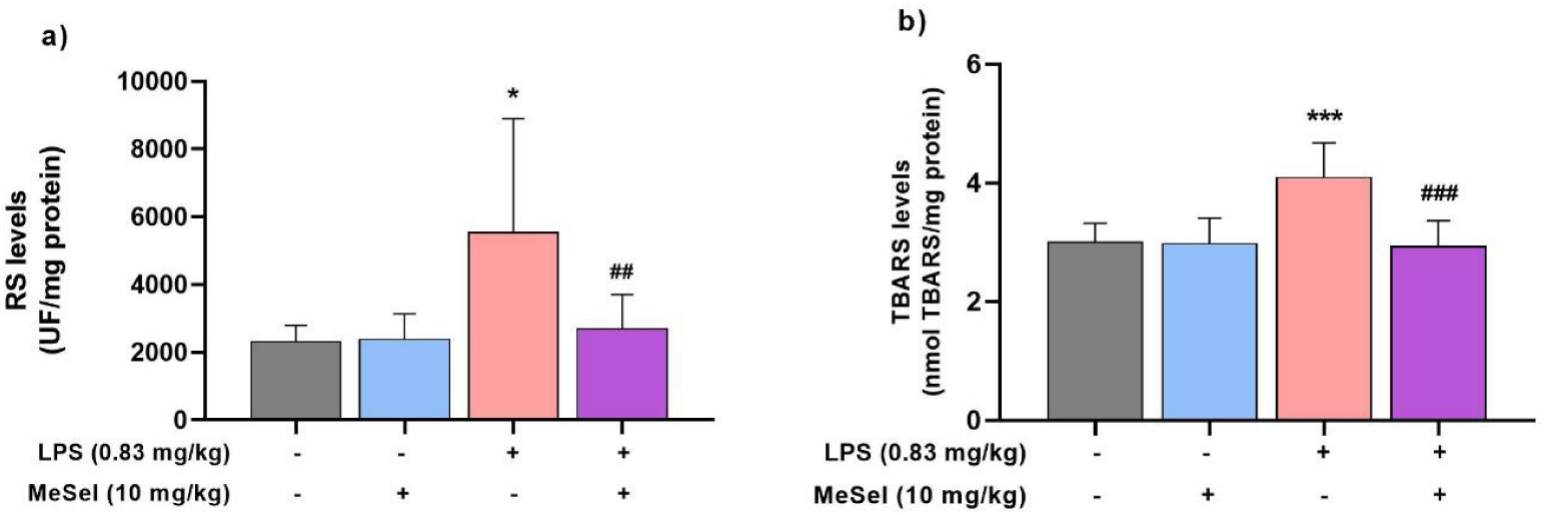
Effect of MeSeI on RS (*F*_3, 27_ = 5.543, *p* = 0.0043) (a) and TBARS (*F*_3, 27_ = 11.68, *p* < 0.0001) (b) levels in the PFC induced by LPS (*n* = 7–8). The values were represented as the mean ± SD and were analyzed using a one-way ANOVA followed by Newman–Keuls post hoc test. (*) *p* < 0.05, (**) *p* < 0.01, and (***) *p* < 0.001 when compared with vehicle group. (^#^) *p* < 0.05, (^##^) *p* < 0.01, and (^###^) *p* < 0.001 when compared with LPS group. Abbreviations: Veh—vehicle; MeSeI—1-(phenylselanyl)-2-(*p*-tolyl)­indolizine; LPS—lipopolysaccharide; RS—reactive species; TBARS—thiobarbituric acid reactive species; PFC—prefrontal cortex.

Excessive RS production and lipidic peroxidation promote the production of more inflammatory cytokines, exacerbating the LPS-induced inflammation. Some RS can further promote intracellular signaling cascades, leading to increased expression of pro-inflammatory genes, such as NF-κB.^26^ MeSeI could prevent these changes, suggesting that the antioxidant potential of MeSeI in the PFC of the mice protected against RS formation and lipid peroxidation induced by LPS. These findings are consistent with the primary astrocyte culture in the first protocol, corroborating the antioxidant properties of MeSeI using different experimental methods.

To continue exploring the possible mechanisms of the action of MeSeI, the protective role of MeSeI against LPS-induced neuroinflammation was examined in PFC through NF-κB and IL-6 mRNA expression levels. The mRNA expression of both biomarkers of neuroinflammation was significantly higher in the PFC of the LPS group mice compared to that in the vehicle one. In the treatment with MeSeI, the mRNA expression of NF-κB (Figure 5a; *F*_3, 8_ = 30.35, *p* = 0.0001) and IL-6 (Figure 5b; *F*_3, 8_ = 49.32, *p* < 0.0001) was significantly reduced in the animals treated with LPS. MeSeI also reduced, *per se,* the NF-κB and IL-6 mRNA. Furthermore, the protein levels of the cytokine IL-6 were significantly higher in the LPS group mice’s PFC than in the vehicle one. In the treatment with MeSeI, the IL-6 levels (Figure 5c; *F*_3, 20_ = 4.485, *p* = 0.0146) were significantly reduced in the animals treated with LPS. These results indicate that MeSeI might relieve the depressive-like behaviors induced by LPS in mice through downregulation of the pro-inflammatory genes.

**Figure 5:**
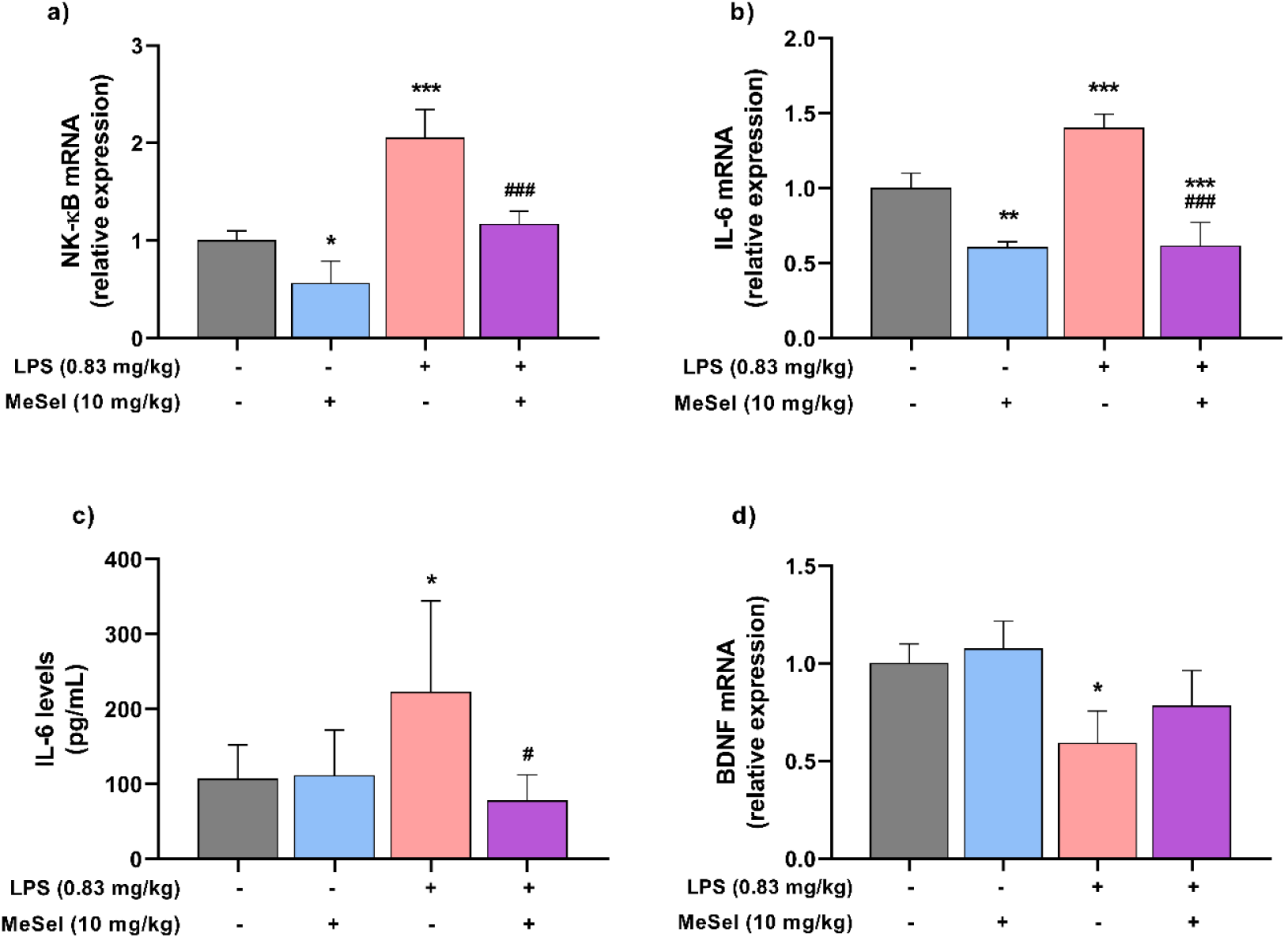
Effects of MeSeI treatment on NF-κB (*F*_3, 8_ = 30.35, *p* = 0.0001) (a) and IL-6 (*F*_3, 8_ = 49.32, *p* < 0.0001) (b) mRNA expression levels, IL-6 protein levels (*F*_3, 20_ = 4.485, *p* = 0.0146) (c), and BDNF (*F*_3, 8_ = 7.164, *p* = 0.0118) (d) mRNA expression levels in the PFC following LPS-induced depression-like behavior in mice (mRNA expression levels *n* = 3; IL-6 protein levels *n* = 6). The values were represented as the mean ± SD and were analyzed using a one-way ANOVA followed by Newman–Keuls post hoc test. (*) *p* < 0.05, (**) *p* < 0.01, and (***) *p* < 0.001 when compared with vehicle group. (^#^) *p* < 0.05 and (^###^) *p* < 0.001 when compared with LPS group. Abbreviations: Veh—vehicle; MeSeI—1-(phenylselanyl)-2-(*p*-tolyl)­indolizine; LPS—lipopolysaccharide; BDNF—brain-derived neurotrophic factor; NF-κB—nuclear factor-kappa B; IL-6—interleukin 6.

In the CNS, LPS initiates an inflammatory cascade by binding to TLR-4, activating transcription factors such as NF-κB, which stimulates the production of inflammatory mediators such as TNF-α, IL-1β, and IL-6.^7,10^ The excessive production of cytokines in the CNS can lead to tissue and cellular damage by hyperstimulation of glial cells. These processes contribute to various depressive symptoms, such as fatigue, anhedonia, and social withdrawal.^9^

The IL-6 contributes to inflammation in acute brain injury, decreases neurogenesis, and promotes stimulation of the hypothalamic–pituitary–adrenal (HPA) axis, leading to increased circulation of glucocorticoids that contribute to the development and persistence of depressive symptoms.^27^ Consequently, suppressing this pathway by reducing NF-κB activation and IL-6 expression could be a key therapeutic approach for neuroprotection and depression treatment. Thus, our results demonstrate that MeSeI can effectively prevent the increase in pro-inflammatory mediators in the PFC of LPS-treated mice, further suggesting its anti-inflammatory potential.

Short stress induced by LPS treatment has detrimental effects on neurogenesis, reducing the production of BDNF, which is responsible for structural and functional cellular support.^28^ As presented in Figure 5d, LPS decreased the expression of BDNF compared to the vehicle group in PFC. However, the MeSeI treatment was not able to significantly prevent this decrease in the MeSeI + LPS group (Figure 5d; *F*_3, 8_ = 7.164, *p* = 0.0118). Possibly, a higher dose of the compound could abrogate this suppression of the BDNF gene since there was a tendency to increase BDNF expression at a dose of 10 mg/kg.

The PFC, hippocampus, and amygdala, due to the high expression of glucocorticoid receptors, can undergo neuronal dysfunction from morphological changes.^29^ Furthermore, dysregulation of the HPA axis can result in the deterioration of astrocytes, affecting both their structure and function. This process contributes to memory deficits observed in depression by diminishing synaptic transmission and plasticity, which is a consequence of disturbed local homeostasis.^19^ The present study showed that there was a significant difference in the levels of corticosterone among the groups (Figure 6; *F*_3, 27_ = 3.605, *p* = 0.0261). Additionally, the study showed that the LPS administration induced depressive-like behaviors accompanied by an increase in plasma corticosterone levels, suggesting HPA axis activation, which was decreased following MeSeI treatment.

**Figure 6:**
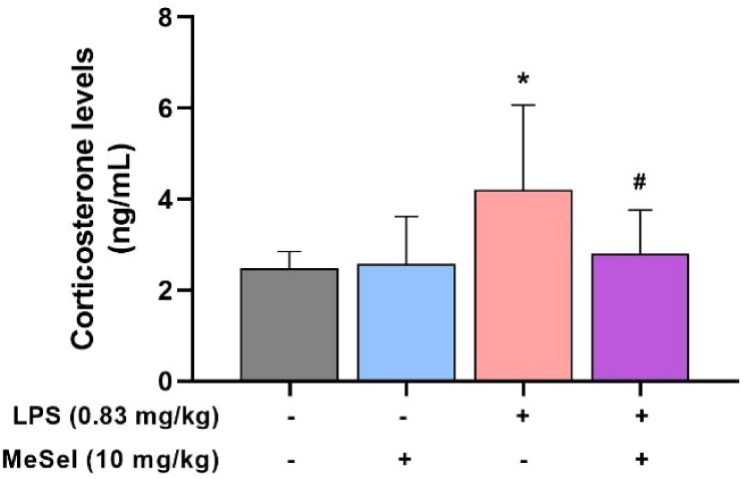
Effect of MeSeI on plasma corticosterone levels induced by LPS (*F*_3, 27_ = 3.605, *p* = 0.0261) (*n* = 7–9). The values were represented as the mean ± SD and were analyzed using a one-way ANOVA followed by Newman–Keuls post hoc test. (*) *p* < 0.05 when compared to the vehicle group. (^#^) *p* < 0.05 when compared to the LPS group. Abbreviations: Veh—vehicle; MeSeI—1-(phenylselanyl)-2-(*p*-tolyl)­indolizine; LPS—lipopolysaccharide.

These findings indicate that MeSeI exerts an antidepressant effect not only through the attenuation of central inflammation and oxidative stress *in vivo* but also via its capacity to protect astrocytes against oxidative damage and promote cellular recovery *in vitro*. Recently, Garcia and coworkers^15^ showed the antioxidant properties of MeSeI *in vitro*, including 1,1-diphenyl-2-picrylhydrazyl (DPPH) scavenger activity and ferric ion (Fe^3+^) reducing antioxidant power (FRAP), as well as assessments of lipid peroxidation and protein carbonylation at low concentrations. Furthermore, our research group demonstrated that MeSeI exerts its antidepressant-like effect in mice through modulation of NMDA glutamate receptors.^14^ This mechanism may be correlated with MeSeI’s neuroprotective effect, as hyperactivation of the glutamatergic system can trigger both neuroinflammation and oxidative stress.^30^

Despite the findings, this study presents some limitations that should be acknowledged. First, it did not include morphological analyses of astrocytes or the expression of astrocytic markers, which would have contributed to a better understanding of astrocyte reactivity. Additionally, further assessments of inflammatory markers, such as NF-κB p65 phosphorylation, apoptosis-related pathways, and activation of the Nrf2/HO-1 pathway analyses, would help to more precisely elucidate the underlying mechanisms involved in the effects of MeSeI on neuroinflammation. Another limitation is that the LPS model was applied exclusively to male mice. Future studies should also evaluate the effects of MeSeI in female mice to account for potential sex-related differences.

In conclusion, these findings indicate that MeSeI exhibits a significant antidepressant-like effect and can minimize symptoms of depression-like behavior in LPS-induced depression in mice. Its main mechanism of action could be the inhibition of activation of NF-κB and reduction of the level of IL-6 in PFC, inhibiting the inflammatory response. Additionally, MeSeI presents antioxidant properties, reducing the RS levels and lipid peroxidation in PFC, along with decreasing plasma corticosterone levels. These findings are complemented by data from primary astrocyte cultures, where MeSeI demonstrated protective effects against oxidative stress, reversing increases in RS and nitrite levels, while restoring sulfhydryl content and improving antioxidant enzyme activities. Taken together, these results suggest that MeSeI might serve as a promising therapeutic candidate for depression by targeting neuroinflammation and restoring redox balance.

## Methods

### Chemicals and Reagents

MeSeI (Figure 7) was prepared and characterized in the Clean Organic Synthesis Laboratory of the Federal University of Pelotas by the method previously described by Penteado and coworkers.^31^ The chemical purity was determined by gas chromatography–mass spectrometry (GC–MS) with a value of 99.9%. Analysis of ^1^H, ^13^C, and ^77^Se NMR spectra showed analytical and spectroscopic data in full agreement with the assigned structure.^12^

**Figure 7:**
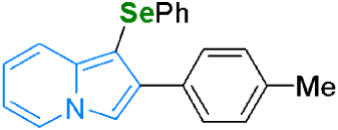
Chemical structure of 1-(phenylselanyl)-2-(*p*-tolyl)­indolizine (MeSeI).

LPS from *Escherichia coli* (serotypes 055:B5 and O127:B8), along with 4-(2-hydroxyethyl) piperazine-1-ethanesulfonic acid (HEPES), sodium bicarbonate (NaHCO_3_), MTT, dimethyl sulfoxide (DMSO), SRB, Coomassie Brilliant Blue G, dichloro-dihydro-fluorescein diacetate (DCFH-DA), and 5,5′-dithiobis (2-nitrobenzoic acid) (DTNB), were sourced from Sigma Chemical Co. (St. Louis, MO, USA). Trichloroacetic acid (TCA) and hydrogen peroxide were purchased from Synth (Brazil). Dulbecco’s modified Eagle’s medium (DMEM), fungizone, penicillin/streptomycin, 0.5% trypsin/ethylenediaminetetraacetic acid (EDTA) solution, and fetal bovine serum (FBS) were obtained from Gibco (Gibco BRL, Carlsbad, CA, USA). FLX was obtained from local suppliers. All other chemicals and solvents employed were of analytical grade.

### Animals

For *in vitro* experiments, newborn Wistar rats (1–2 days old), male or female, were utilized, while for *in vivo* experiments, adult Swiss male mice (weight 25–30 g) were employed. All animal procedures were approved by the Ethics Committee on the Use of Animals of the Federal University of Pelotas with protocol numbers 31292 and 030811. The animals were kept at a controlled temperature (22 ± 2 °C) with a 12 h light/dark cycle and had free access to food and water. The use of the animals was designed to minimize animal suffering according to the NIH guidelines for the care and use of laboratory animals (NIH publication n° 8023, revised 1978) and the ARRIVE Guidelines for Reporting Animal Research.

### Protocol 01

#### Primary Astrocyte Culture

This *in vitro* model is widely accepted for evaluating neuroinflammatory mechanisms and is highly responsive to LPS stimulation (Figure 8). Primary astrocyte cells were isolated from newborn Wistar rats as previously described by da Frota and coworkers.^32^ The cerebral cortices were mechanically minced and dissociated with calcium- and magnesium-free balanced salt solution (pH 7.4), consisting of 137 mM NaCl, 5.36 mM KCl, 0.27 mM Na_2_HPO_4_, 1.1 mM KH_2_PO_4_, and 6.1 mM glucose (CMF). The resulting cell suspension was centrifuged at 1000 g for 10 min, and the pellet was resuspended in DMEM supplemented with 10% FBS (pH 7.6). Identity as astrocytes was confirmed based on morphological criteria and consistency with previously established protocol.^32^ For cytotoxicity assays and oxidative stress analysis, 3 × 10^5^ and 3 × 10^4^ cells were seeded in poly-l-lysine-coated 6-well and 96-well plates, respectively. Astrocytes were cultured in a CO_2_ incubator at 37 °C for 20 days, with medium changes every 5 days.

**Figure 8:**
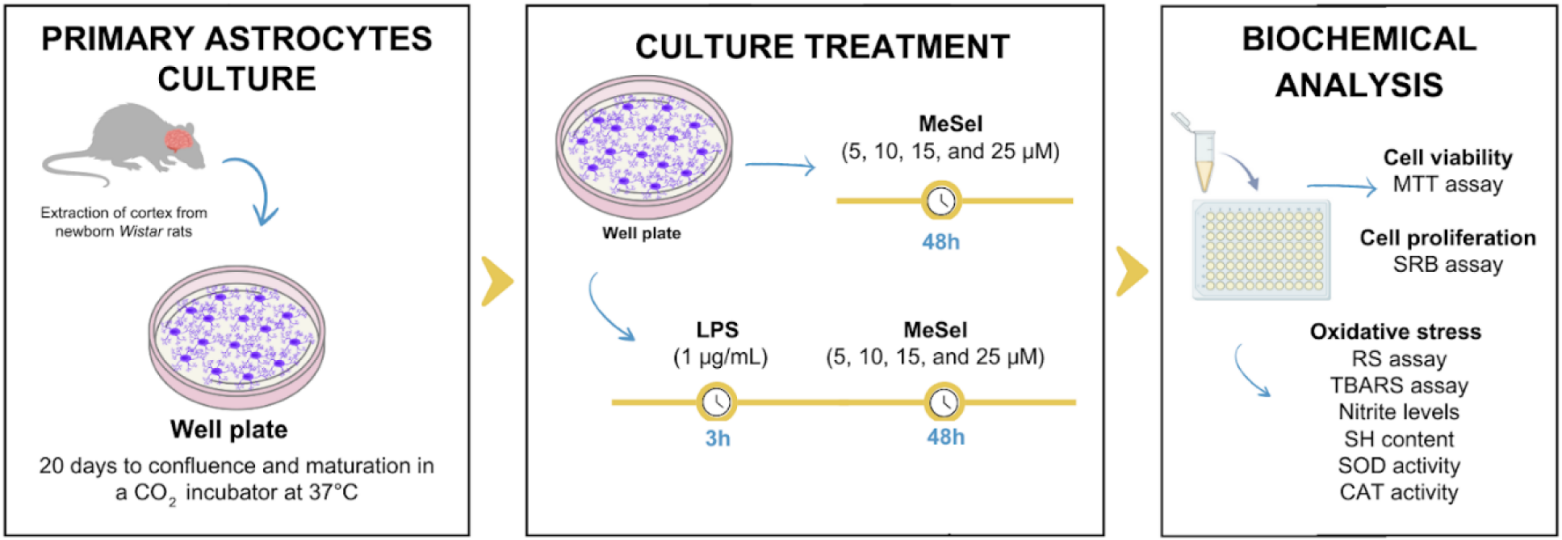
Experimental design for the effects of LPS and MeSeI on primary astrocyte culture. Abbreviations: MeSeI—1-(phenylselanyl)-2-(*p*-tolyl)­indolizine; LPS —lipopolysaccharide; MTT—diphenyl tetrazolium bromide; SRB—sulforhodamine B; RS—reactive species; TBARS—thiobarbituric acid reactive species; SH—sulfhydryl; SOD—superoxide dismutase; CAT—catalase. Created using Canva.com.

#### Cell Culture Treatment with LPS and MeSeI

MeSeI was dissolved in 0.05% DMSO and then mixed with DMEM containing 10% FBS to achieve solutions at final concentrations of 5, 10, 15, and 25 μM. To assess cytotoxicity, astrocytes were exposed to four different concentrations of MeSeI for 48 h. To evaluate the neuroprotective potential of MeSeI, the astrocytes were exposed to LPS as described by Alvez and coworkers.^33^ Briefly, cells were first exposed to LPS (1 μg/mL) for 3 h and then treated with MeSeI alone for 48 h (Figure 8). Control cells were maintained in 0.05% DMSO.

#### Cytotoxicity

##### Cell Viability

Cell viability was measured using the MTT assay, which assesses viable cells by detecting the reduction of yellow tetrazolium MTT to blue formazan through the action of dehydrogenase enzymes in active mitochondria.^34^ Following the treatments, the cultures were washed with CMF and incubated with an MTT solution (0.5 mg/mL per well) at 37 °C in a humidified 5% CO_2_ atmosphere for 90 min. Subsequently, the medium was removed, and the formazan crystal products were dissolved in DMSO. Finally, optical density (OD) was measured at 492 nm using a microplate reader (SpectraMAX 190). Results were expressed as a percentage of vehicle by using the following formula: Cell viability rate (%) = (OD of treated cells/OD of vehicle) × 100%.

##### Cell Proliferation

Cell proliferation was measured by the SRB assay, based on the determination of cell protein content.^35^ Following the treatments, the culture medium was removed, and the culture was washed and fixed in 50% TCA and incubated at 4 °C for 45 min. Subsequently, the cells were washed five times with distilled water. Next, 0.4% SRB was added, and the cultures were incubated in the dark at room temperature for 30 min. After incubation, the cells were then washed five times with 1% acetic acid to remove noncomplexed dye from the proteins, and SRB was eluted with 10 mM Tris. Finally, the OD was measured at 530 nm using a microplate reader (SpectraMax190). Results were expressed as a percentage of vehicle using the following formula: Cell proliferation rate (%) = (OD of treated cells/OD of vehicle) × 100%.

### Protocol 02

#### LPS-Induced Depressive-Like Behavior in Mouse Model

The ability of MeSeI to prevent depressive-like behavior induced by LPS treatment was investigated in this experiment. For this, MeSeI was dissolved in canola oil and administered intragastrically (i.g.) at a dose of 10 mg/kg in mice, based on a previous study by our group.^12^ LPS, used to induce a depressive-like state in mice, was dissolved in saline solution (0.9%) and administered intraperitoneally (i.p.) at a dose of 0.83 mg/kg.^36^ Finally, FLX was included in the study to validate the behavioral tests. FLX was dissolved in saline solution (0.9%) and administered by the i.p. route at a dose of 20 mg/kg.^37^

#### Experimental Design

The experimental design was separated into two sets, according to Figure 2b. The mice were randomly divided into five groups in each set (*n* = 7–9 per group): (1) vehicle group (canola oil, 10 mL/kg, i.g.; saline, 10 mL/kg, i.p.); (2) the MeSeI group (MeSeI, 10 mg/kg, i.g.; saline, 10 mL/kg, i.p.); (3) LPS group (canola oil, 10 mL/kg, i.g.; LPS, 0.83 mg/kg, i.p.); (4) MeSeI plus LPS group (MeSeI, 10 mg/kg, i.g.; LPS, 0.83 mg/kg, i.p.); and (5) FLX plus LPS group (FLX, 20 mg/kg, i.p.; LPS, 0.83 mg/kg, i.p.).

First, mice were treated with canola oil (10 mL/kg, i.g.), MeSeI (10 mg/kg, i.g.), or FLX (20 mg/kg, i.p.), and after 30 min, LPS (0.83 mg/kg, i.p.) or saline (10 mL/kg, i.p.) was injected. Twenty-four hours after the final LPS administration, behavioral tests (first set: OFT followed by FST; second set: TST followed by ST) were carried out on the sets of mice. Subsequently, the animals were anesthetized with isoflurane for blood collection by a cardiac puncture and then euthanized. Brain tissue was removed to isolate the PFC (first set: biochemical analyses; second set: molecular analyses).^38,39^ All samples were stored at −80 °C for later use (Figure 9).

**Figure 9:**
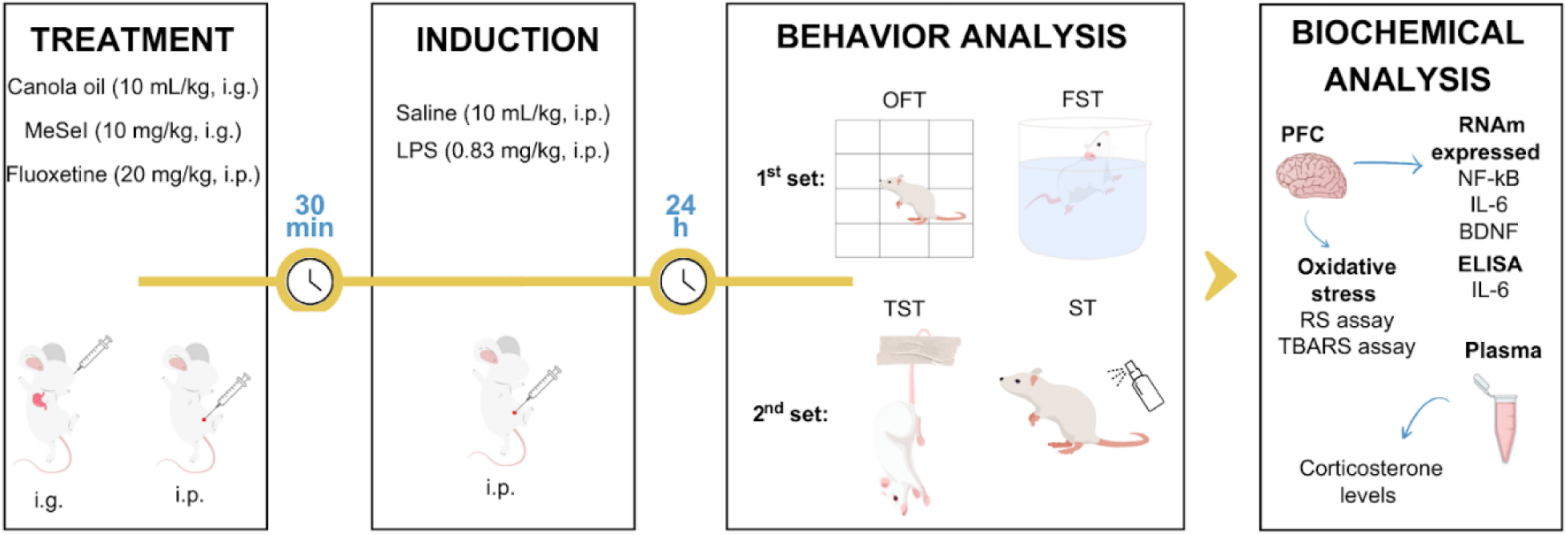
Experimental design for the evaluation of MeSeI effects on LPS-induced depression-like behavior in mice. Abbreviations: MeSeI—1-(phenylselanyl)-2-(*p*-tolyl)­indolizine; LPSlipopolysaccharide; i.g.—intragastric; i.p.—intraperitoneal; OFT—open field test; FST—forced swimming test; TST—tail suspension test; ST—splash test; PFC—prefrontal cortex; ELISA—enzyme-linked immunosorbent assay; NF-κB—nuclear factor-kappa B; IL-6—interleukin 6; BDNF—brain-derived neurotrophic factor; RS—reactive species; TBARS—thiobarbituric acid reactive species. Created using Canva.com.

#### Behavior Analysis

Based on the literature, the most commonly used tests—FST, TST, and ST—were selected to assess the depressant-like behavior induced by LPS in mice. In addition, OFT was performed to evaluate the animal’s locomotor activity.^38,39^ These behavioral tests were performed 24 h after the administration of LPS in two sets. In the first set, the OFT was performed, followed by FST. In the second set, mice were submitted to TST and ST. Behavioral test analysis was performed by an investigator who was blinded to avoid potential bias. Animals that presented behavioral anomalies, such as tail climbing during the tail suspension test and diving during the forced swimming test, were excluded from the study.

##### Open Field Test (OFT)

The evaluation of the locomotor and exploratory activity of the mice was carried out using the OFT in order to rule out any effect of possible locomotor or exploratory alterations caused by treatment with the MeSeI.^40^ The mice were placed in the center of a wooden box (30 × 30 × 15 cm) divided into nine squares of equal areas, and for 4 min, the number of squares crossed (locomotor activity) and the number of rearings (exploratory activity) were evaluated. Between each mouse testing, the arena was cleaned with 20% ethanol afterward to prevent residual olfactory traces.

##### Forced Swimming Test (FST)

The FST allows the antidepressant-like behavior in animals through the analysis of immobility time and the absence of escape-oriented behavior. The mice were placed individually in a cylinder (10 cm in diameter by 25 cm in height) containing 19 cm of water at 25 ± 1 °C. The total test time is 6 min, but the immobility time was recorded only in the last 4 min, as the first 2 min were used for the animal’s habituation. Animals were considered immobile when they remained floating motionless in the water or made movements to keep their nose above the water surface.^41^

##### Tail Suspension Test (TST)

The TST, with some modifications,^42^ was performed to measure despair behavior, one of the symptoms of depression. Each mouse was individually suspended 50 cm above the floor with adhesive tape placed approximately 1 cm from the tip of the tail. The immobility time in this condition was recorded during the last 4 min of a 6-min session, in which the first 2 min served only to habituate the animal. The definition of immobility is the absence of escape behavior displayed by the animal.

##### Splash Test (ST)

ST allows self-care behavior to be assessed as motivational behavior, according to Birmann and coworkers.^43^ This method allows the spraying of a 10% sucrose solution onto the dorsal coat of mice in a cage. Due to the viscosity of this solution, the animals begin grooming behavior, such as body, nose, and face grooming and head washing. Grooming behavior can be considered to be an indirect measure of the hedonic state in mice. The total test time is 5 min, and during this interval, the latency to the first grooming episode and the time spent grooming were recorded. During the test, the mouse stayed in a cage, and after each measurement, the apparatus was cleaned with 20% ethanol spray.

### Biochemical Evaluation

#### Sample Preparation

For *in vitro* experiments, cell lysates were prepared for oxidative stress assays. After 48 h of treatment, the cultures were washed twice with sterile water, and cell lysates were manually prepared using a cell scraper. The samples collected were then centrifuged at 1000 rpm for 10 min. The pellet was discarded, and the supernatant was used for subsequent biochemical analysis.

For *ex vivo* experiments, the animals were anesthetized with isoflurane inhalation for blood collection by cardiac puncture and then euthanized, and the PFC was quickly removed. PFC tissue from the first experimental set was collected and homogenized in 50 mM Tris-HCl, pH of 7.4 (1:10, w/v). The homogenates were centrifuged at 900 × *g* for 10 min at 4 °C. The supernatants were used to determine RS levels and lipid peroxidation. The PFC from the second experimental set was immersed in Trizol and maintained at −80 °C to perform the real-time quantitative polymerase chain reaction (RT-PCR). The blood sample obtained from the second experimental set via cardiac puncture was transferred to heparinized tubes. After centrifugation (10 min at 2500 × *g*), the plasma was stored at −80 °C to determine corticosterone levels.

#### Reactive Species (RS) Measurement

RS production was determined using the methods described by Loetchutinat and coworkers.^44^ In cell cultures, RS levels were measured based on the reaction of dichloro-dihydro-fluorescein diacetate (DCFH-DA) with intracellular RS to produce a fluorescent intermediate, 2″,7″-dichlorofluorescein (DCF). Cultures were incubated for 30 min at 37 °C with 1 μM DCFH-DA, and fluorescence was recorded at 485/520 nm. RS levels were expressed as μmol of DCF/mg of protein.

In the PFC of mice, RS levels were quantified by incubating homogenate samples with Tris-HCl (10 mM, pH 7.4) and 0.0033 mM DCHF-DA. The presence of RS oxidized DCHF-DA to the fluorescent DCF. Thus, the fluorescence intensity of DCF was recorded at 520 nm, and excitation was recorded at 488 nm in a spectrophotometer. Values were expressed as units of fluorescence (UF)/mg of protein.

#### Lipid Peroxidation Assessment

Lipid peroxidation in lysates of astrocytes and PFC was measured by the TBARS assay. This way, the TBARS test was performed to determine the damage to lipid membranes caused by RS. For the test, an aliquot of the homogenized supernatant was incubated with 8.1% sodium dodecyl sulfate (SDS), 0.8% TBA, and acetic acid/HCl (pH 3.4) at 95 °C for 1 h. Lastly, the TBARS levels were measured spectrophotometrically at a wavelength of 532 nm. The results were expressed as nmol TBARS/mg protein.^45^

#### Nitrite Levels

Nitrite production was assessed in lysates of astrocytes following the method of Stuehr and Nathan.^46^ For this reaction, cell culture supernatants were incubated with 1% sulfanilamide for 10 min at room temperature. The samples were then mixed with the Griess reagent (0.1% *N*-[1-naphthyl]­ethylenediamine dihydrochloride) and incubated in the dark for another 10 min. OD was measured at 540 nm using a microplate reader (SpectraMax190), and a standard curve of sodium nitrate was used to determine the nitrite levels in the samples.

#### Total Sulfhydryl (SH) Content

The total SH content in astrocyte lysates was measured using the DTNB assay as described by Aksenov and Markesbery.^47^ This method is based on the reduction of DTNB by thiols, producing a yellow derivative, 5′-thio-2-nitrobenzoic acid (TNB), whose absorbance was measured at 412 nm and correlated with the SH content. The SH content was expressed as nanomoles of TNB/mg of protein.

#### Superoxide Dismutase (SOD) Activity

SOD activity in lysates of astrocytes was evaluated using the method described by Misra and Fridovich.^48^ This assay measures the inhibition of superoxide-dependent adrenaline autoxidation, with SOD scavenging superoxide anions. OD was measured at 480 nm using a microplate reader (SpectraMax 190). Results were expressed as units per milligram of protein.

#### Catalase (CAT) Activity

CAT activity was measured using the method described by Aebi,^49^ which is based on the decomposition of hydrogen peroxide (H_2_O_2_) in potassium phosphate buffer (pH 7.0). The reaction was monitored at 240 nm by using a microplate reader (SpectraMax 190) at 37 °C. CAT activity was expressed as units per milligram of protein.

#### Protein Determination

The protein level of the samples was determined according to the methodology of Bradford^50^ and Lowry and coworkers,^51^ using bovine serum albumin as a standard (1 mg/mL).

#### Plasma Corticosterone Measurement

Plasma corticosterone levels were determined according to Zenker and Bernstein.^52^ The diluted plasma samples in distilled water (2:8) were treated with chloroform and subsequently submitted to a washing process using NaOH (0.1 M) to remove the solvent. In the extraction step, the fluorescence reagent (sulfuric acid-ethanol, 7:3, v/v) was used, and then the acidic phase was incubated in the dark for 2 h. Finally, plasma corticosterone levels were determined by fluorescence in a fluorimeter (247 nm excitation and 540 nm emission wave), and the results were expressed in ng/mL.

#### Enzyme-Linked Immunosorbent Assay (ELISA)

Cytokine IL-6 was estimated in PFC homogenate by using a commercially available ELISA kit for mouse IL-6 (RAB0308, 0929J0413, Sigma-Aldrich, MO, USA). The PFC samples were homogenized in a buffer solution (50 mM Tris-HCl, pH of 7.4) and then centrifuged at 900 × *g* for 10 min at 4 °C. The supernatant was separated for IL-6 estimation. The pro-inflammatory cytokine IL-6 level in PFC samples was quantified using the ELISA kit according to the manufacturer’s instructions.

#### Extraction of Messenger RNA and Gene Expression by qRT-PCR

According to the manufacturer’s instructions, the total RNA of PFC tissue was extracted from the samples using a TRIZOL (Invitrogen, Carlsbad, CA). Next, the total RNA was treated with RNase-free DNase (Invitrogen, Carlsbad, CA). The 0.1% formaldehyde agarose gel was used to assess the samples’ quality. After, RNA was quantified spectrophotometrically. The primers of the NF-κB, IL-6, and BDNF used for the real-time PCRs were synthesized by Invitrogen (São Paulo, Brazil) (Table 1). The amplification reaction for real-time PCR was conducted utilizing SYBR Green One-Step qRT-PCR with Rox (Invitrogen, Carlsbad, CA), following the manufacturer’s guidelines. cDNA synthesis was performed using 0.5 μg of total RNA and gene-specific forward and reverse primers (20 μM) for each gene. PCR reactions were run in a 7500 Real-Time Fast thermocycler (Applied Biosystems) with the following conditions: 50 °C for 15 min, 95 °C for 52 min, followed by 40 cycles at 95 °C for 15 s and 60 °C for 30 s. A dissociation curve step was carried out at 95 °C for 5 min with a final step at 4 °C. This entire assay was conducted for each gene and encompassed cDNA from both treated samples and the control without a template.

**Table 1:** Primer Sequence for qRT-PCR^a^

Genes	Forward	Reverse
NF-κB	GCTTTCGCAGGAGCATTAAC	CCGAAGCAGGAGCTATCAAC
IL-6	AGAGATACAAAGAAATGATGGA	AGCTATGGTACTCCACAAGACCA
BDNF	CCATAAGGACGCGGACTTGTAC	AGACATGTTTGCGGCATCCAGG
β-actin	AGAGGGAAATCGTGCGTGAC	CAATAGTGATGACCTGGCCGT

a Abbreviations: NF-κB—nuclear factor-kappa B; IL-6—interleukin 6; BDNF—brain-derived neurotrophic factor.

The results were quantified in terms of CT (threshold cycle) values. CT is calculated by the software, which sets a threshold line at the baseline fluorescent signal and identifies the data point that intersects with this threshold. The CT value is inversely related to the initial template copy number. To assess the variations in CT values among the control group, the treated group, and the endogenous control β-actin gene for each reaction (ΔCT), the 2−ΔΔCT method was utilized. The relative expression levels of the genes postincubation were determined by dividing the expression units of the treated group by those of the control group. All measurements were conducted in duplicate with 4 animals per group. The results were expressed as relative concentration calculated as described by Giongo and coworkers.^53^

## Statistical Analysis

The data were presented as mean ± SD (standard deviation). The statistical difference was determined by one-way analysis of variance (ANOVA) followed by Newman–Keuls multiple comparisons post hoc test when groups achieved normality by D’Agostino–Pearson test. The value of *p* < 0.05 was considered to be significant. The data were analyzed using GraphPad Prism 8.0.2 software (GraphPad, San Diego, CA, USA). The N of the experiments was validated by G*Power version 3.1.9.7 (Franz, Universität Kiel, Germany).

## Abbreviations

BDNF: brain-derived neurotrophic factor
CAT: catalase
CNS: central nervous system
DCF: 2″,7″-dichlorofluorescein
DCFH-DA: dichloro-dihydro-fluorescein diacetate
DMEM: Dulbecco’s modified Eagle’s medium
DMSO: dimethylsulfoxide
DPPH: 1,1-diphenyl-2-picrylhydrazyl
DTNB: 5,5′-dithiobis (2-nitrobenzoic acid)
EDTA: trypsin/ethylenediaminetetraacetic acid
FBS: fetal bovine serum
FLX: fluoxetine
FRAP: ferric ion (Fe^3+^) reducing antioxidant power
FST: forced swimming test
GC–MS: gas chromatography–mass spectrometry
H_2_O_2_: hydrogen peroxide
HEPES: piperazine-1-ethanesulfonic acid
HPA: hypothalamic–pituitary–adrenal
i.g.: intragastrically
i.p.: intraperitoneally
IL: interleukin
LPS: lipopolysaccharide
MAO: monoamine oxidase
MeSeI: 1-(phenylselanyl)-2-(*p*-tolyl)­indolizine
MTT: 3-(4,5-dimethylthiazol-2-yl)-2,5-diphenyl tetrazolium bromide
NaHCO_3_: sodium bicarbonate
NMDA: *N*-methyl-d-aspartate
NF-κB: nuclear factor-kappa B
NO: nitric oxide
Nrf-2: erythroid 2-related factor 2
OD: optical density
OFT: open field test
PFC: prefrontal cortex
RS: reactive species
RT-PCR: real-time quantitative polymerase chain reaction
SDS: sodium dodecyl sulfate
SH: sulfhydryl
SOD: superoxide dismutase
SRB: sulforhodamine B
SSRI: selective serotonin reuptake inhibitor
ST: splash test
TBARS: thiobarbituric acid reactive species
TCA: trichloroacetic acid
TLR: toll-like receptors
TNB: 5′-thio-2-nitrobenzoic acid
TNF: tumor necrosis factor
TST: tail suspension test
WHO: World Health Organization
